# Updates on the distribution and diversity of sand flies (Diptera: Psychodidae) in Romania

**DOI:** 10.1186/s13071-019-3507-7

**Published:** 2019-05-20

**Authors:** Cristina Daniela Cazan, Ioana Raluca Păstrav, Angela Monica Ionică, Gizem Oguz, Ozge Erisoz Kasap, Vit Dvorak, Petr Halada, Mirabela Oana Dumitrache, Petr Volf, Bulent Alten, Andrei Daniel Mihalca

**Affiliations:** 1 Department of Parasitology and Parasitic Diseases, Faculty of Veterinary Medicine, University of Agricultural Sciences and Veterinary Medicine Cluj-Napoca, Cluj-Napoca, Romania; 2 Department of Biology, Ecology Section, Faculty of Science, VERG Laboratories, Hacettepe University, Ankara, Turkey; 3 Department of Parasitology, Faculty of Science, Charles University, Prague, Czech Republic; 4 Institute of Microbiology of the Czech Academy of Sciences, Prague, Czech Republic

**Keywords:** Sand flies, Distribution, Diversity, Canine leishmaniasis, Romania

## Abstract

**Background:**

Phlebotomine sand flies (Diptera: Psychodidae) are haematophagous insects that transmit the protozoan parasite *Leishmania infantum* (Kinetoplastida: Trypanosomatidae), the main causative agent of both zoonotic visceral leishmaniasis (VL) and canine leishmaniasis (CanL) in the Mediterranean basin. Eight species of sand flies have been previously recorded in Romania: *Phlebotomus papatasi*, *Phlebotomus alexandri*, *Phlebotomus sergenti*, *Phlebotomus perfiliewi*, *Phlebotomus neglectus*, *Phlebotomus longiductus*, *Phlebotomus balcanicus* and *Sergentomyia minuta.* Three of them (*P. perfiliewi*, *P. neglectus* and *P. balcanicus)* were incriminated as vectors of *L. infantum.* Recent reports of autochthonous CanL in Romania require updates on sand fly distribution and diversity in this country.

**Methods:**

Between 2013–2014 and 2016–2018, CDC light traps and mouth aspirators were used to collect sand flies in 132 locations from Romania, indoors and around various animal species shelters. Species identification of collected specimens was done using morphological keys, genetic tools and MALDI-TOF protein profiling.

**Results:**

Sand flies were present in seven localities (5.3%): Eibenthal, Baia Nouă, Gura Văii (south-western Romania, Mehedinţi County); Fundătura, Pâhneşti, Epureni (eastern Romania, Vaslui County); and Schitu (southern Romania, Giurgiu County). Of the total number of collected sand flies (*n *= 251), 209 (83.27%) were *Phlebotomus neglectus*, 39 (15.53%) *P. perfiliewi*, 1 (0.40%) *P. papatasi*, 1 (0.40%) *P. balcanicus* and 1 (0.40%) *P. sergenti* (sensu lato).

**Conclusions:**

We confirmed the presence of five sand fly species previously recorded in Romania. However, their updated distribution differs from historical data. The diversity of sand fly species in Romania and their presence in areas with Mediterranean climatic influences constitutes a threat for the reemergence of vector-borne diseases. In the context of CanL and VL reemergence in Romania, but also due to imported cases of the diseases in both humans and dogs, updates on vector distribution are imperative.

**Electronic supplementary material:**

The online version of this article (10.1186/s13071-019-3507-7) contains supplementary material, which is available to authorized users.

## Background

Phlebotomine sand flies (Diptera: Psychodidae) are haematophagous insects demonstrated to transmit the protozoan parasite *Leishmania infantum*, the main causative agent of both zoonotic visceral leishmaniasis (VL) and canine leishmaniasis (CanL) in the Mediterranean region [1]. Sand flies are of high importance for both veterinary and public health because of their role as vectors for leishmaniases and other bacterial and viral diseases [2, 3].

Of approximately 25 sand fly species present in Europe, several species were proven or suspected of *Leishmania* transmission, mostly of the genus *Larroussius* (6 species) and *Adlerius* (1 species) [3]. Of these, three vectors of VL and CanL caused by *L. infantum* were recorded in Romania: *Phlebotomus* (*Larroussius*) *perfiliewi* Parrot, 1930; *Phlebotomus* (*Larroussius*) *neglectus* Tonnoir, 1921; and *Phlebotomus* (*Adlerius*) *balcanicus* Theodor, 1948. Other species were also recorded in the country: *Phlebotomus* (*Phlebotomus*) *papatasi* (Scopoli, 1786); *Phlebotomus* (*Paraphlebotomus*) *alexandri* Sinton, 1928; *Phlebotomus* (*Paraphlebotomus*) *sergenti* Parrot, 1917; *Phlebotomus* (*Adlerius*) *longiductus* Parrot, 1928; and *Sergentomyia* (*Sergentomyia*) *minuta* (Rondani, 1843). Their historical distribution and diversity in Romania were reported by various authors between 1910 and 1971 [4–20].

As no later update regarding the species composition of sand flies is available in Romania our study aimed to bring new data on their distribution and diversity. The need of updated knowledge of the vector distribution is more urgent after the recent reemergence of autochthonous VL and CanL [21, 22], followed by the demonstration of seropositivity in dogs [23]. Moreover, new cases of imported CanL cases in Romania were reported [24, 25] which raised the question of possible incrimination of locally available, vector competent species in the trigger of further local transmission.

## Methods

### Study area and design

Between 2013–2014 and 2016–2018, 267 CDC miniature light traps (Trappola per Monittoraggio Zanzare, IMT Original 2002, Italy and John W. Hock, model 512, Gainesville, FL, USA) were placed in 132 localities in 21 counties in Romania (see Additional file 1: Table S1), during the expected activity of sand flies (July-August). One to eight traps were set in each locality per night. In some localities, more than one trapping site was selected, according to the visual evaluation of the field conditions and the possible occurrence of sand flies, considering their environmental requirements. A standardized protocol was used [26]. Traps were placed over night (19:00–5:30 h), in and around animal shelters, close to the walls, at about 1.5 m height. The traps were placed in locations selected according to the known habitat preferences of sand flies: altitude below 750 m, old stables, with decomposing organic matter, abundant dejections, walls built on a wood structure with a composition of clay, dry grass or stables with large agglomerations of domestic animals, abandoned buildings of former stables and ruins, along with old fences built with rocks [27]. In general, nights with no wind and no precipitations were chosen for sampling. Geographical coordinates, animal-related and shelter-related data were recorded on site. In one location (Eibenthal, Mehedinţi County), sand flies were also collected directly from the walls by mouth aspirator (John W. Hock Company, USA).

### Species identification

After each trapping night, the sand flies were separated from other insects, and stored in 70% ethanol. For species identification, heads and genitalia of each specimen were dissected and slide-mounted in Swan solution, previously cleared with Marc-André solution (chloral hydrate/acetic acid). The sand flies were morphologically identified using entomological key [28] relying on specific morphological features of the pharynx and genitalia (spermathecae in females, external genitalia in males).

As the morphological identification of female specimens of species within the subgenera *Larroussius* and *Adlerius* may be challenging, molecular techniques were also applied for species confirmation. Seventeen specimens morphologically identified as different species (five *P. perfiliewi*, five *P. neglectus*, one *P. papatasi*, one *P. sergenti* sensu lato (s.l.) and specimens without a robust identification [four *Phlebotomus* (*Larroussius*) spp., one *Phlebotomus* (*Adlerius*) spp.] were randomly selected for DNA barcoding analysis. Additionally, other eight specimens belonging to *P. perfiliewi* morphospecies were subjected to MALDI-TOF protein profiling. The method was chosen as a new, time- and cost-effective molecular approach for species identification to help robustly identify *P. perfiliewi* females which can be challenging for morphological identification due to close resemblance with other *Larroussius* species, namely *P. tobbi*.

The DNA was extracted individually from the thorax of each specimen using the Qiagen DNeasy Blood and Tissue Kit (Qiagen, Austin, Texas, USA) following the manufacturer’s instructions and stored at − 20 °C. PCR amplifications of the cytochrome *c* oxidase subunit 1 (*cox*1) gene region (~ 660 bp) were performed in 50 µl reaction volume using LCO1490 and HCO2198 primers [29]. The amplification products were separated and visualized on 2% agarose gels, purified using the QIAquick PCR Purification Kit (Qiagen) and directly sequenced in both directions using the primers used for DNA amplification (ABI Prism BigDye Terminator Cycle Sequencing Ready Reaction Kit, Foster City, USA). Sequences were edited and aligned using BioEdit v.7.0.9.0 [30]. To construct taxon identity trees, obtained sequences and the similar sequences deposited in GenBank were subjected to Neighbor Joining (NJ) analysis using the Kimuraʼs 2- parameter (K2P) nucleotide substitution model in MEGA6.0 [31].

For MALDI-TOF protein profiling, thoraxes with wings and legs were manually homogenized, proteins were extracted from the homogenate by 25% formic acid and spotted on steel target plates with a matrix of an aqueous 60% acetonitrile/0.3% TFA solution of sinapinic acid (30 mg/ml; Sigma-Aldrich, St. Louis, USA) as previously described [32]. Protein mass spectra were measured on an Ultraflex III MALDI-TOF spectrometer (Bruker Daltonics, Bremen, Germany) within a mass range of 3–25 kDa and with external calibration using the Bruker Protein Calibration Standard I. Each spectrum represented an accumulation of 1000 laser shots (20 × 50 laser shots from different positions of the sample spot). For species identification, obtained protein profiles were processed using MALDI Biotyper 3.1 and searched against our in-house database which currently comprises reference spectra of 23 different sand fly species. Log score value (LSV) > 2.0 was accepted as the unambiguous assignment.

### Mapping

The maps were generated using QGis 2.18.0 software (http://www.qgis.org). For historical data (presence/absence), georeferenced records were extracted from previously published data [23].

## Results

**Fig. 1:**
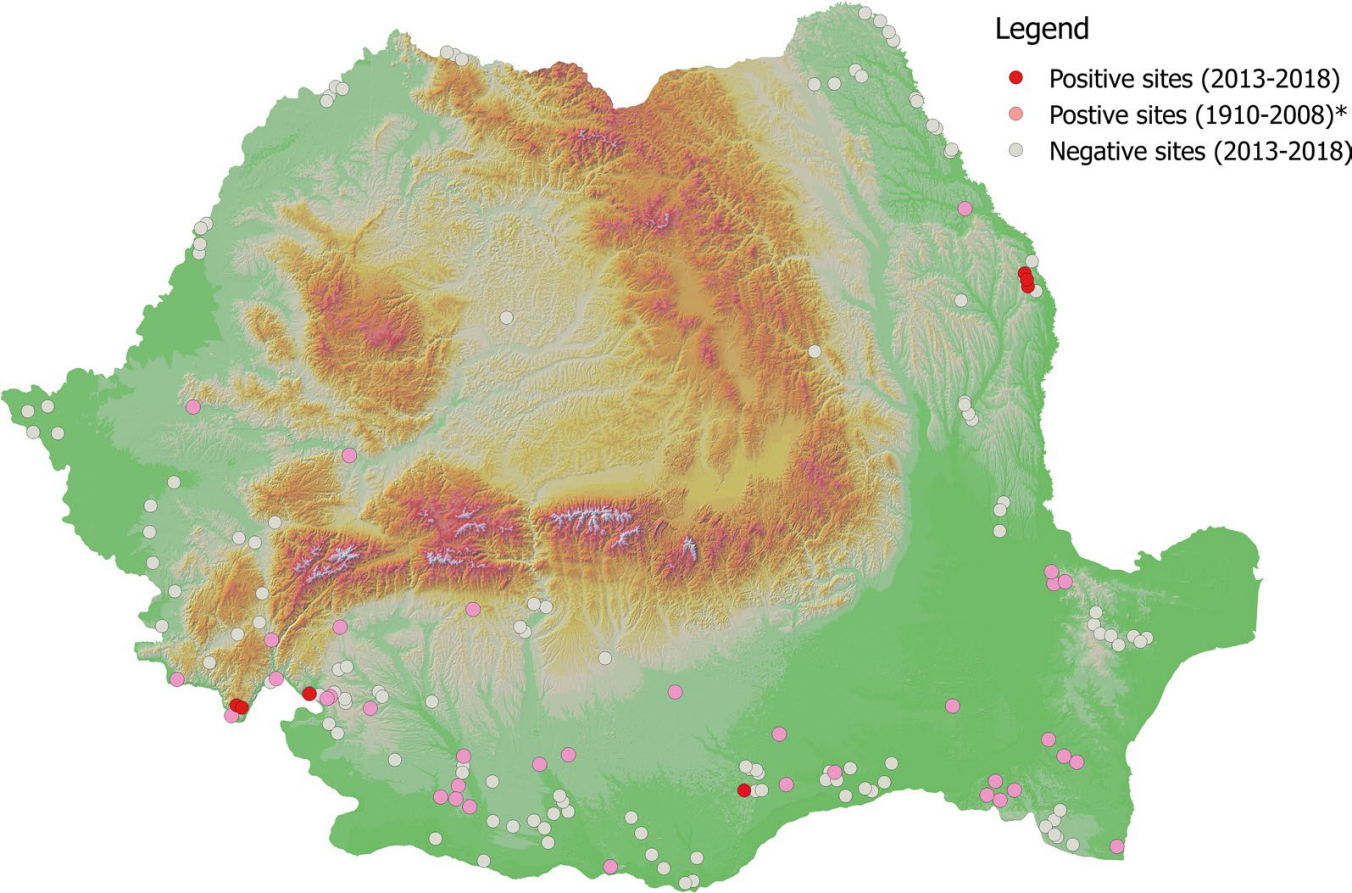
All positive and negative sites for all sand fly species. Asterisk indicates historical data [23]

Sand flies were present in 7 out of the 132 sampled localities (5.3%). The positive and negative localities are shown in Fig. 1. A total of 251 sand flies (33 males and 218 females) were collected between 2013–2014 and 2016–2018. Out of 218 females collected, 42 (19.27%) were blood-fed and 5 (2.30%) were gravid. All specimens belonged to genus *Phlebotomus* (subgenera *Phlebotomus, Paraphlebotomus, Adlerius*, and *Larroussius*) (see Additional file 1: Table S1). The total number of positive localities for each species varied from one to five (Table 1).

**Table 1:** Trapped sand fly species by locality, sex, gravidity status and feeding status in Romania (2013–2014; 2016–2018)

	*P. neglectus*	*P. perfiliewi*	*P. papatasi*	*P. balcanicus*	*P. sergenti* s.l.
Collected specimens	209/251 (83.27%)	39/251 (15.53%)	1/251 (0.40%)	1/251 (0.40%)	1/251 (0.40%)
Positive localities	3/132 (2.30%)	5/132 (3.80%)	1/132 (0.75%)	1/132 (0.75%)	1/132 (0.75%)
Collected males	32/209 (15.31%)	1/39 (2.60%)	0/1 (0%)	0/1 (0%)	0/1 (0%)
Collected females	177/209 (84.69%)	38/39 (97.40%)	1/1 (100%)	1/1 (100%)	1/1 (100%)
Blood-fed females	42/177 (23.70%)	0/38 (0%)	0/1 (0%)	0/1 (0%)	0/1 (0%)
Un-fed females	135/177 (76.30%)	38/38 (100%)	1/1 (100%)	1/1 (100%)	1/1 (100%)
Gravid females	3/177 (1.70%)	1/38 (2.64%)	0/1 (0%)	0/1 (0%)	1/1 (100%)
Non-gravid females	174/177 (98.30%)	37/38 (97.36%)	1/1 (100%)	1/1 (100%)	0/1 (0%)

**Fig. 2:**
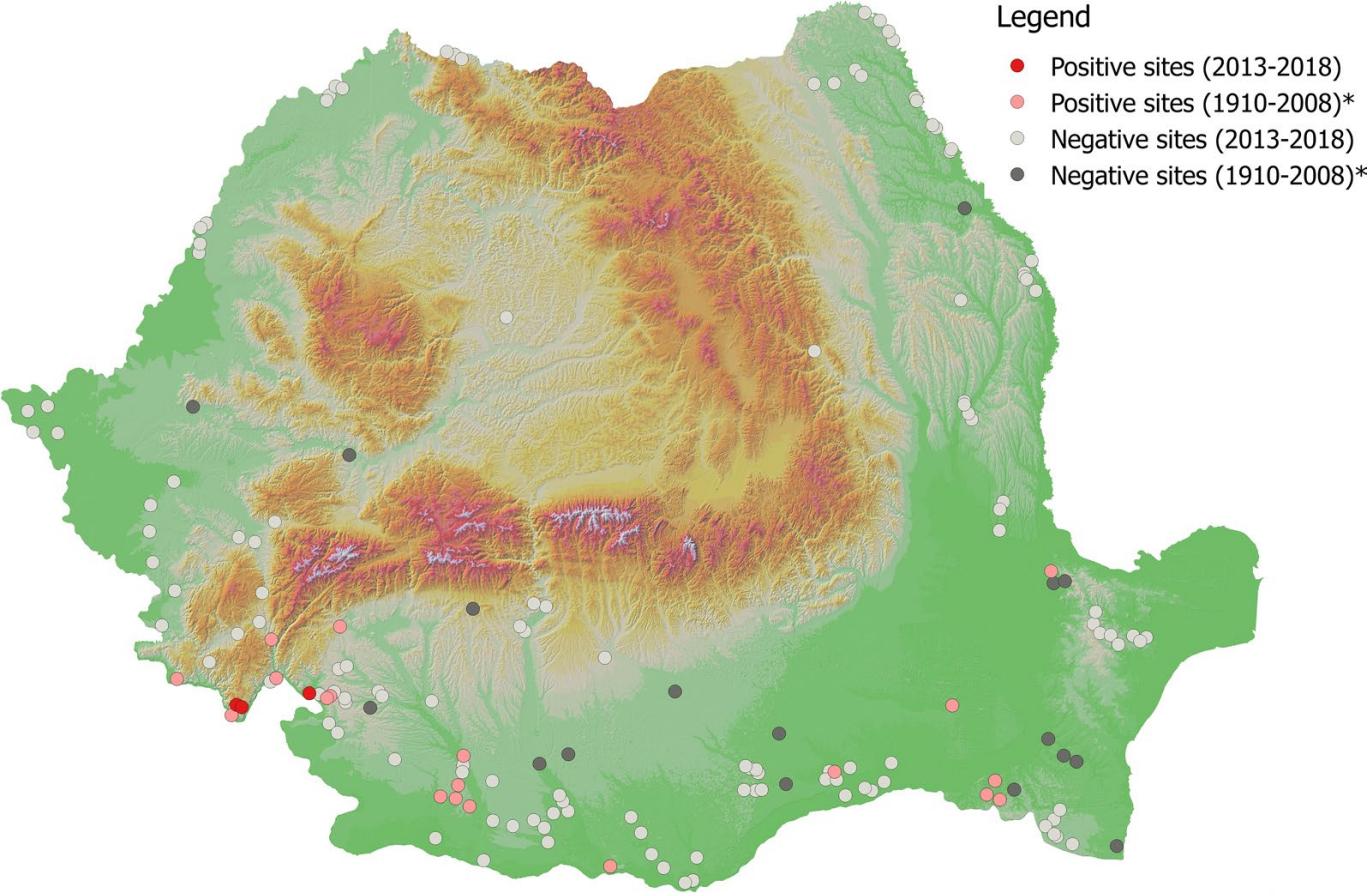
Distribution of *P. neglectus*. Asterisk indicates historical data [23]

**Fig. 3:**
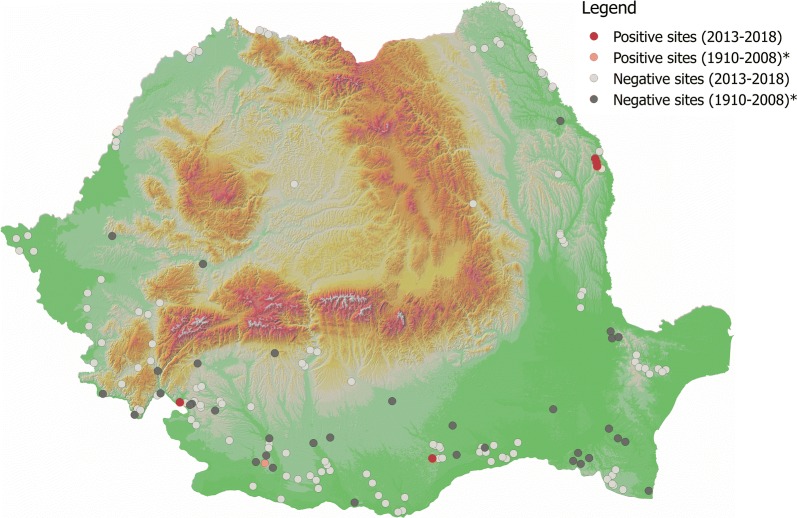
Distribution of *P. perfiliewi*. Asterisk indicates historical data [23]

**Fig. 4:**
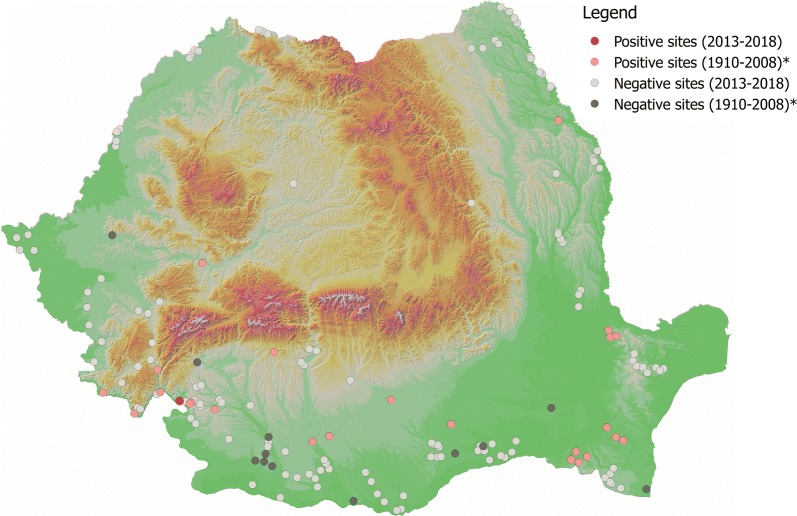
Distribution of *P. papatasi*. Asterisk indicates historical data [23]

**Fig. 5:**
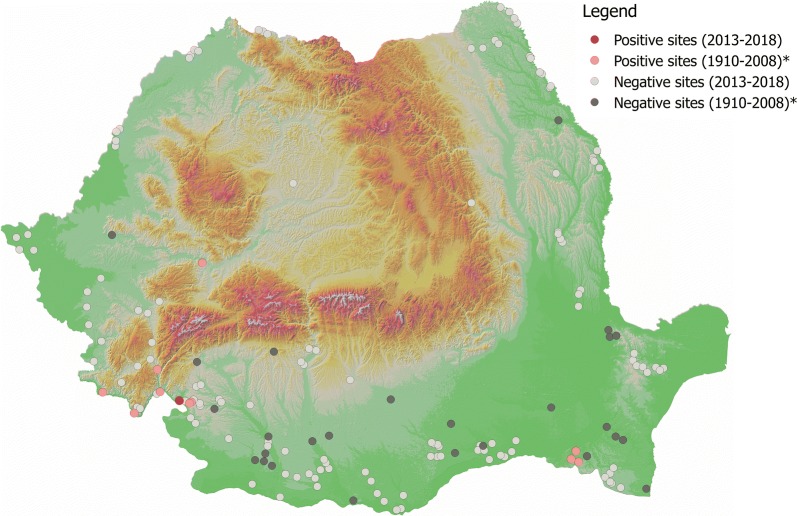
Distribution of *P. balcanicus*. Asterisk indicates historical data [23]

**Fig. 6:**
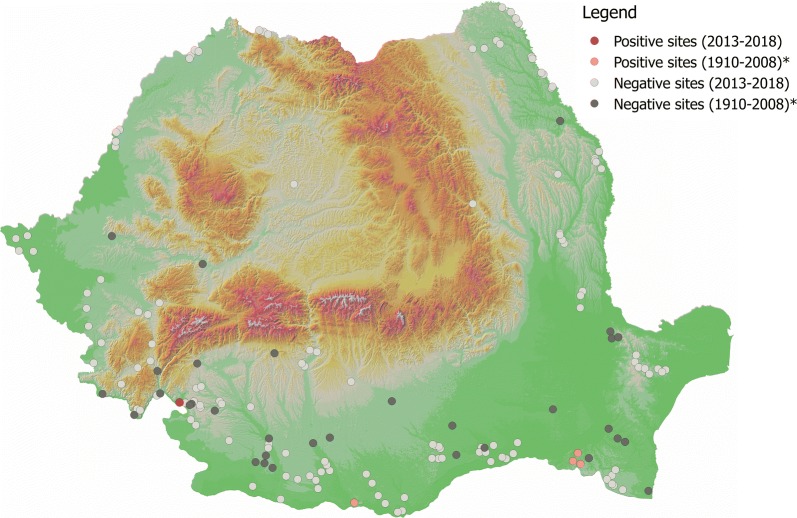
Distribution of *P. sergenti* s.l. Asterisk indicates historical data [23]

Two species, *P. neglectus* and *P. perfiliewi* were dominant (83.27% and 15.53%, respectively). *Phlebotomus neglectus* was found in Eibenthal, Baia Nouă and Gura Văii (south-western Romania, Mehedinţi County). *Phlebotomus perfiliewi* was found in Gura Văii (south-western Romania, Mehedinţi County), Fundătura, Pâhneşti, Epureni (eastern Romania, Vaslui County), and Schitu (southern Romania, Giurgiu County). Other three species, *P. papatasi*, *P. balcanicus* and *P. sergenti* s.l., were represented by a single specimen in a single locality (Gura Văii, Mehedinţi County, Romania). The present data (presence/absence) as well as historical (presence/absence) data for each of the five species identified in our study are shown in Figs. 2, 3, 4, 5 and 6.

**Fig. 7:**
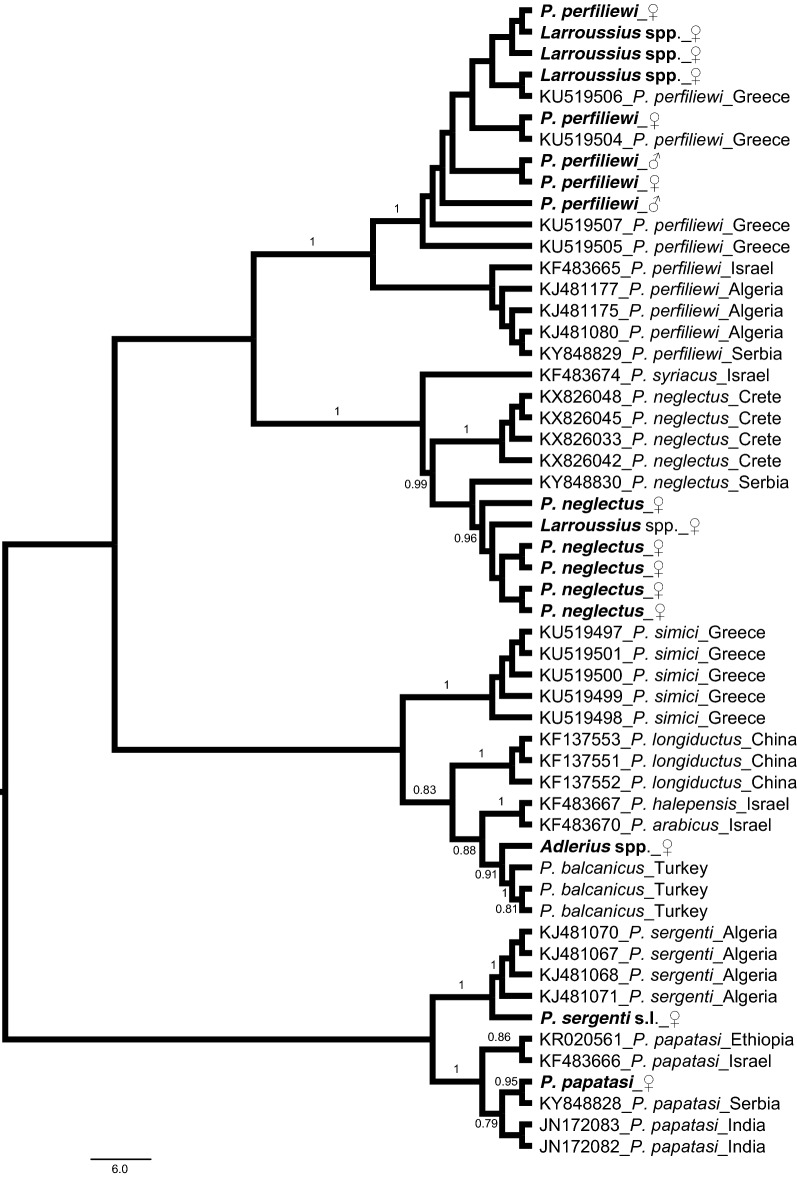
Neighbor joining tree constructed using the mitochondrial *cox*1 sequences of sand fly species from Romania (tip labels in bold) and sequences obtained from GenBank. Bootstrap values higher than 70% are shown

The aligned *cox*1 sequences of Romanian sand fly species were found to be 610 bp long without any deletions, insertions or stop codons. NJ analysis of these sequences with the ones available in GenBank and in our database showed that the specimens belonging to the same species clustered together. In addition, three *Larroussius* spp. females were assigned as *P. perfiliewi* and one as *P. neglectus*. The only specimen belonging to the subgenus *Adlerius* was grouped with *P. balcanicus* from Turkey (E. Kasap & B. Alten, unpublished data). Although the mean K2P distance between the Turkish and Romanian specimens was found to be high (5.3%), this difference could be attributed to the geographical origins of the individuals examined (Fig. 7). For the eight *P. perfiliewi* and five *P. neglectus* sequences analysed, only two and three unique haplotypes were detected, respectively. The sequence data of the haplotypes obtained for all the Romanian sand fly species are available in the GenBank database under the accession numbers MK425631–MK425638.

**Fig. 8:**
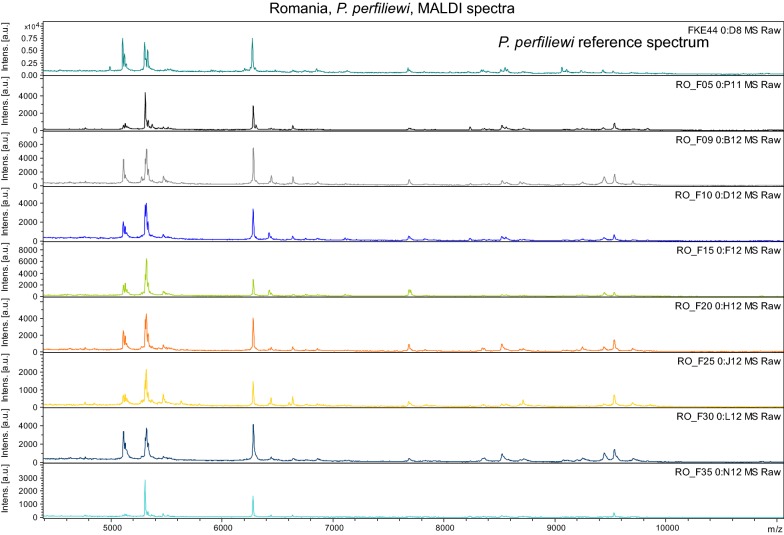
MALDI spectra for *P. perfiliewi* (origin Romania)

All specimens analysed by MALDI-TOF protein profiling were identified as *P. perfiliewi* (Fig. 8). Obtained spectra showed good quality and scored over a threshold of LSV 2, except for one specimen (RO_F35) which produced a spectrum of compromised quality and LSV < 2, nevertheless also for this specimen *P. perfiliewi* was the first suggested identification. Figure 8 shows overall protein spectra compared with a reference spectrum of *P. perfiliewi* from our in-house database.

## Discussion

In the present study, five *Phlebotomus* species were identified out of the eight sand fly species previously described in Romania [33]. Three of them are of high importance in terms of *L. infantum* transmission: *P. neglectus*, *P. perfiliewi* and *P. balcanicus* [3]. Two of these three species represented 98.80% of all collected sand flies in the present study, highlighting the risk for local diseases transmission foci, as recent autochthonous and imported cases of VL and CanL were reported [21, 22, 24, 25].

*Phlebotomus neglectus* was the most abundant species in the present study (83.27%). It belongs to the *P. major* complex which currently comprises six species with different distribution: *P. major* in India, Nepal and Pakistan; *P. wui* in China; *P. notus* in Afghanistan; *P. wenyoni* in Iran and Iraq; *P. syriacus* in south-western part of Asia and Caucasia; and *P. neglectus* in south and south-eastern regions of Europe and Crimea) [34]. The species was reported for the first time in Romania in 1957 [13]. Its presence was historically recorded along the Danube valley and Bărăgan plain (south-western, southern and south-eastern Romania). It was mostly present outdoor, in arid to humid natural sites, at altitudes of 200–300 m, in valleys and at caves entrance, in crevices, but also peri-domestically, on building walls. The recorded temperature of its activity in Romania was between 24.9–29.8 °C, with average annual temperature of 11 °C [11, 15]. The current distribution of the species is limited to Mehedinţi Plateau (south-eastern Romania), at similar latitudes and environmental conditions as previously historically described [11, 15].

*Phlebotomus neglectus* has an important role as a vector for *L. infantum* in south-central, southern and eastern Europe and its presence and distribution was mostly correlated with CanL surveillance, in countries like Greece [35], Albania [36], Croatia [37], Montenegro [38] or Hungary [39]. In these countries, the species is also the most abundant and found at latitudes between 35.63°N (Karpathos Island, Greece), the southernmost, and 47.43°N, the northernmost (Törökbálint, Hungary). These geographical territories comprise regions with Mediterranean climate (Greece, Albania, Croatia and Montenegro), its transition to the temperate climate (northern Greece) and temperate climate with Mediterranean influences (southern Hungary and southern Romania).

The second most abundant species of the present study was *P. perfiliewi.* In Romania, the presence of *P. perfiliewi* was mentioned for the first time in 1956 [12]. The species was suspected for the first time to transmit VL and CanL in Romania in 1971 [11]. Its presence in Romania was described between the 10 °C and 11 °C isotherms [12]. In the present study, *P. perfiliewi* was present in five out of seven positive sites in south-western, southern and north-eastern Romania, the latter being a new locality, outside its previously known range. The species seems to have the widest geographical distribution in the country. It is also present in the neighbouring countries at similar average annual temperatures and latitudes [39–41].

Only one specimen of each *P. papatasi*, *P. balcanicus* and *P. sergenti* s.l. was collected from the same site, Gura Văii, Mehedinți County (south-western Romania). *Phlebotomus papatasi* was mentioned for the first time more than 100 years ago in Iaşi, north-eastern Romania [4]. In has also been found in parts of central, south-western, southern and south-eastern Romania [15, 16]. *Phlebotomus papatasi* is the vector for *Leishmania major* in North Africa and the Middle East and it is also known to transmit several phleboviruses [3]. The species is distributed from Portugal and Morocco in the West to India in the East [28]. In Serbia, a neighbouring country of Romania, *P. papatasi* was the predominant species in a recent study from 2017 [41]. In the present study, *P. papatasi* appears to have a more restricted distribution than previously described [15, 16] (Fig. 4).

*Phlebotomus balcanicus* was mentioned for the first time in Romania in 1958 [14]. The species was present in the central, south-western and southern regions of the country [11, 18]. Its current known distribution is limited to the Balkans, Turkey, Armenia and Georgia [41, 42]. Its current known distribution in Romania is limited to the south-eastern region of the country.

*Phlebotomus sergenti* s.l. was found in the southern and south-eastern parts of Romania [11]. The species is distributed in the semi-arid regions of the Mediterranean basin, North Africa, and Middle East, mostly at altitudes between 0–200 m [3, 43]. In the present study, its distribution in Romania is represented by south-eastern parts of the country.

The highest diversity of sand fly species recorded in the present study comprising all five collected species was in Mehedinţi Plateau (south-eastern Romania). Compared to the other positive locations situated in southern and north-eastern regions, where only one species was present, Mehedinţi Plateau has some distinctive climatic characteristics: (i) it is the only geographical region in Romania that has Mediterranean climatic influences (rainy autumns and softer winters); (ii) the annual average temperatures are between 10–11 °C, with 2–3 °C higher compared to the rest of the country; (iii) the altitudes are between 400–600 m, with precipitations between 700–800 mm [44]. Significant correlations were described between such climatic parameters (latitude, altitude, annual average temperature), the first date of sand fly collection and the type of the abundance trend [42], or between a higher species diversity and altitudes of around 900 m [43].

## Conclusions

The updates provided by the present study on the distribution and diversity of the sand fly species in Romania show changes in both parameters, compared to historical data. Most localities where sand flies were reported at the beginning of the 20th Century are nowadays negative. This could be explained by a series of factors like the widespread use of insecticides in Romania during the malaria eradication programmes (1958–1964) [33], or the alterations of the sand fly habitats as a consequence of environmental, demographic, human behavioural factors or climate changes in the last decades [3]. Also, three other species that appeared to be present in the country, *P. alexandri*, *P. longiductus* and *S. minuta*, were not present in the surveyed trapping sites in our study. The negative trapping sites do not exclude, though, their presence in the country as the study has its limitations (e.g. more than one team to work in the field, nonhomogeneous data collection). The diversity of sand fly species in Romania and their presence in areas with Mediterranean climatic influences constitutes a threat for the reemergence of vector-borne diseases and a continuous surveillance is recommended in order to monitor the multiannual dynamics and to understand the real sand fly diversity and distribution. In the context of CanL and VL re-emergence, but also due to imported cases of the diseases in both humans and dogs, updates on vector distribution are crucial.

## Additional file

**Additional file 1: Table S1.** Sampling information, site codes and trapped sand flies in Romania (2013–2015; 2016–2018).

## Data Availability

The datasets supporting the conclusions of this article are included within the article and its additional files. The sequence data of the haplotypes obtained for all the Romanian sand fly species are available in GenBank database under the accession numbers MK425631–MK425638.

## Abbreviations

VL: visceral leishmaniasis
CanL: canine leishmaniasis
CDC: Centre of Disease Control and prevention
MALDI-TOF: matrix-assisted laser desorption/ionization time of flight mass spectrometry
LSV: log score value
s.l.: sensu lato
*cox*1: cytochrome *c* oxidase subunit 1 gene
NJ: neighbor joining
